# Exploring the role of peripheral nerves in trauma-induced heterotopic ossification

**DOI:** 10.1093/jbmrpl/ziae155

**Published:** 2024-11-22

**Authors:** Clifford T Pereira, Sean H Adams, K C Kent Lloyd, Trina A Knotts, Aaron W James, Theodore J Price, Benjamin Levi

**Affiliations:** Department of Surgery, University of California, Davis School of Medicine, Sacramento, CA 95816, United States; Department of Surgery, University of California, Davis School of Medicine, Sacramento, CA 95816, United States; University of California, Davis Center for Alimentary and Metabolic Science, Davis, CA 95816, United States; Department of Surgery, University of California, Davis School of Medicine, Sacramento, CA 95816, United States; University of California, Davis Center for Alimentary and Metabolic Science, Davis, CA 95816, United States; Department of Surgery, University of California, Davis School of Medicine, Sacramento, CA 95816, United States; University of California, Davis Center for Alimentary and Metabolic Science, Davis, CA 95816, United States; Department of Pathology, John’s Hopkins University, Baltimore, MD 21287, United States; Department of Neuroscience, Center for Advanced Pain Studies, University of Texas at Dallas, Richardson, TX 75080, United States; University of Texas, Southwestern Medical Center, Dallas, TX 75080, United States

**Keywords:** neural inflammation, heterotopic ossification, trauma, peripheral nerves, progenitor cells, signaling pathway

## Abstract

Recent studies have linked pain and the resultant nociception-induced neural inflammation (NINI) to trauma-induced heterotopic ossification (THO). It is postulated that nociception at the injury site stimulates the transient receptor potential vanilloid-1 (the transient receptor potential cation channel subfamily V member 1) receptors on sensory nerves within the injured tissues resulting in the expression of neuroinflammatory peptides, substance P (SP), and calcitonin gene-related peptide (CGRP). Additionally, BMP-2 released from fractured bones and soft tissue injury also selectively activates TRVP1 receptors, resulting in the release of SP and CGRP and causing neuroinflammation and degranulation of mast cells causing the breakdown the blood-nerve barrier (BNB), leading to release of neural crest derived progenitor cells (NCDPCs) into the injured tissue. Parallel to this process BMP-2 initiates the NCDPCs toward osteogenic differentiation. CGRP has direct osteogenic effects on osteoprogenitor cells/mesenchymal stem cells, by activating BMP-2 via canonical Wnt/β-catenin signaling and cAMP-cAMP-response element binding protein signaling. BMP-2 binds to TGF-βRI and activates TGF-β-activated kinase 1 (TAK1) leading to phosphorylation of SMAD1/5/8, which binds to the co-activator SMAD4 and translocates to the nucleus to serve as transcription factor for BMP responsive genes critical in osteogenesis such as *Runx2* and others. Thus, NINI phenotypes, and specifically CGRP induction, play a crucial role in THO initiation and progression through the activation of the BMP pathway, breakdown of the BNB, leading to the escape of NCDPCs, and the osteogenic differentiation of the latter.

## Introduction

Heterotopic ossification (HO) is the aberrant formation of extra-skeletal bone within soft tissues and can be secondary to a genetic etiology, such as fibrodysplasia ossificans progressive (FOP)^1^; or acquired from traumatic etiology such as traumatic brain injury, spinal cord injury, burns, blast injuries, and surgeries such as joint replacements.^1^^,^^2^ For the purposes of this review article, we will focus on trauma-induced heterotopic ossification (THO), which has a 10%-20% incidence after injury.^3^ THO is thought to involve a multy-step process: (1) tissue injury and inflammation, followed by (2) bone formation. Bone formation occurs in 3 stages: (1) angiogenesis (endothelial and lymphatic) and hyperproliferation of mesenchymal stem cells (MSCs)/mesodermal progenitor cells (MPCs), (2) chondrogenic differentiation of MSCs/MPCs, and (3) osteogenesis (intramembranous and endochondral ossification). Once developed THO can cause recurrent skin breakdown, infections, and joint immobility that require recurrent surgeries and hospitalizations. The aberrant bone also causes severe pain and opioid dependence is common. In the case of amputees, prosthetic fitting and use are painful due to subcutaneous bone in weight-bearing regions, and this can ultimately lead to prosthetic abandonment.^3^

Despite being first recognized since the early 1800s, there are currently no effective prophylactic modalities to prevent debilitating condition or any interventions to resorb HO once it has formed. Nonsteroidal anti-inflammatory drugs such as indomethacin and celecoxib (typically for 3-6 months) are used in high-risk patients such as those undergoing hip arthroplasty, but the optimal dosage and duration are still being determined.^4^ Low-dose radiation, corticosteroids, and bisphosphonates are utilized for prophylaxis, of which radiation is the preferred approach for orthopedic surgeons. There is currently only 1 FDA-approved bisphosphonate—etidronate, for THO prevention but this has poor long-term efficacy.^2^ All these prophylactic therapies unfortunately have prohibitive side effects, especially in pediatric populations, which include impaired bone and wound healing, renal and electrolyte disturbances, cardiovascular and gastrointestinal complications, and the theoretical risk of cancer in the case of radiation therapy.^4^ Surgical excision is the mainstay of treatment and is only recommended after completion of osseous maturation that usually occurs after 6 months. Even after surgery is performed, complete HO excision is never achieved given the diffuse nature of the bone that involves muscle and subcutaneous tissue or bony tissue that encases neurovascular structures. Incomplete surgical resection is associated with a 27% rate of recurrence. Furthermore, the trauma of surgery itself can further promote recurrence.^5^ Thus, THO is a devastating and debilitating condition without any effectual prophylactic or treatment modalities. A better understanding of the initiation and progression of THO is required to enable development of therapeutics targeting the complex biological processes that underlie the condition.

The nervous system is divided into the central nervous system (CNS) consisting of the brain and spinal cord; and the peripheral nervous system (PNS) consisting of the somatic (sensory and motor nerves), and the autonomic nervous system (ANS). The PNS has long been implicated in THO formation. For instance, THO has a higher incidence with surgeries or injuries near nerves such as in the elbow or hip. Fracture dislocations of elbows tend to stretch the ulnar nerve and have a 15%-37% higher incidence of THO compared with other fractures in the upper extremity. Similarly fracture dislocations of the hip, and hip joint surgeries where the common surgical techniques (such as lateral or anterolateral approaches to the hip joint, dislocation of the hip, trochanteric osteotomies, resection of the femoral head, etc.) stretch the sciatic nerve and are associated with a significantly higher risk of THO.^3^^,^^5^ Compared with historic data from previous wars, incidence and severity of THO has significantly increased in injured veterans from the Iraq and the Afghanistan wars. Traumatic limb amputations in combat have a 60%-70% incidence of THO formation, which increases to over 80% if the amputation was performed within the zone of injury.^3^ This increased incidence is believed to occur due to the higher explosive weaponry and higher survival due to advancements in body armor, evacuation, and frontline medical treatment when compared with prior conflicts.^3^ Civilian high-impact traumatic amputations have a 94% incidence of radiographic evidence of THO and a 22.5% incidence of symptomatic THO. This increased incidence of THO in high-impact injuries such as blast injuries has recently been linked to pain, and sensory nerves within the zone of injury.^6^^,^^7^ Recently, THO has been clinically associated with neuroma formation in amputees and is also noted to occur in a rodent neuroma model. Thus, the PNS has long been clinically associated with THO formation. While recent review articles have focused on the molecular/mechanistic basis of THO,^2^ this article is an update on our understanding of the peripheral nerve influences on THO formation, and our current gaps in knowledge that require further research. We also discuss potential therapeutic avenues for the treatment of THO and THO-induced pain.

## Nociception and nociception-induced neural inflammation

**Figure 1:**
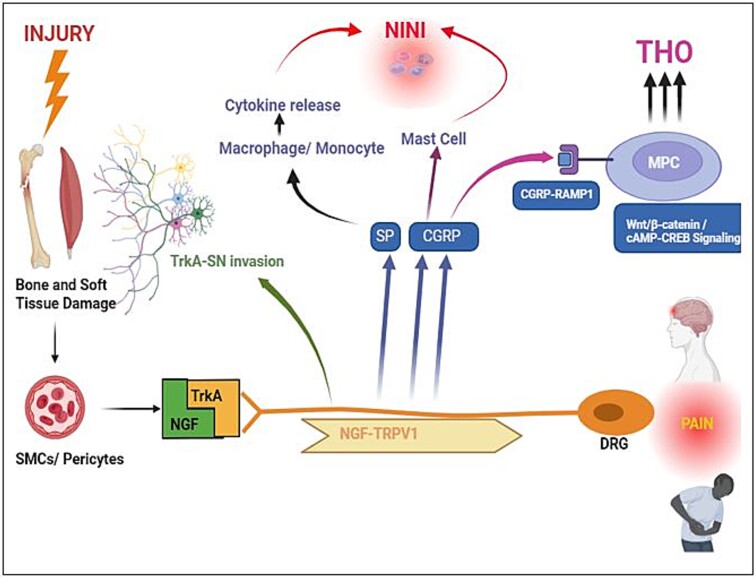
Traumatic injury causes pain mediated by nerve growth factor (NGF) expressed by vascular smooth muscles (SMCs) and pericytes promoting the expression of factors that activate inflammation and osteogenesis. NGF activates the transient receptor potential vanilloid-1 (TRPV1) channels on sensory nerves within the injured tissue by binding to its receptor—tropomyosin receptor kinase A. Once activated the NGF-TRPV1 complex is internalized within the sensory axon through endocytosis and is transported retrograde to the dorsal root ganglion cell body leading to pain hypersensitivity. TRPV1 activation also initiates nociception-induced neural inflammation (NINI), which triggers the secretion of substance P (SP) and calcitonin gene-related peptide (CGRP). CGRP binds to its receptor activity modifying protein (RAMP1) on tissue-resident mesodermal progenitor cells (MPCs) and activates osteogenic differentiation via multiple intracellular signaling cascades including canonical Wnt/β-catenin signaling and cAMP-cAMP-response element binding protein (CREB) signaling.

Traumatic injuries cause pain, and a central mediator of this pain is nerve growth factor (NGF), which is expressed in injured tissue from several cell types such as vascular smooth muscles and pericytes.^6^ NGF acts on sensory nerves responsible for the detection of injurious stimuli, called nociceptors, within the injured tissue by binding to its receptor—tropomyosin receptor kinase A (TrkA), which in turn activates intracellular signaling pathways such as hydrolysis of intracellular phosphatidylinositol 4,5-bisphosphate, leading to sensitization of ion channels responsible for detecting noxious stimuli, like transient receptor potential vanilloid-1 (TRPV1).^7^ Transient receptor potential receptors are a family of ion channels that consist of 28 members grouped into 6 families. Of these, the TRP vanilloid (TRPV) are thermoactivated and consists of 6 members, ie, TRPV1 to TRPV6. Of these TRPV1 receptor is best known as it is the primary receptor responsible for the sensing of noxious heat and is a highly specific receptor for capsaicin. TRPV1 integrates stimuli from many cell types including being activated by cell damage and death and it is a primary receptor thought to produce pain and hyperalgesia after injury.^8^ Once activated the NGF-TRPV1 complex is internalized within the sensory axon through endocytosis and is then transported retrograde to the dorsal root ganglion cell body via signaling endosomes, leading to sustained signaling that can change the transcriptional landscape of the injured nociceptor. This retrograde signaling can cause increased TRPV1 expression through the activation of p38, a regulator of pro-inflammatory cytokines such as TNFα and IL-1β.^9^ Additionally, the injury-induced NGF expression drives the progressive invasion of TrkA-expressing sensory neurons and sympathetic nerves into the injury site (Figure 1). We have shown that surgical denervation impedes axonal ingrowth preventing bone formation within the injured tissue.^10^^,^^11^ Single cell sequencing in these studies showed a shift from TGFβ to FGF signaling following the loss of neural stimulation driving the signaling away from chondrogenesis and osteogenesis.^10^^,^^11^ Similarly, a reduction in THO has been demonstrated using transgenic THO-animal models lacking TRPV1 receptors or with interrupted NGF-TrkA signaling.^6^^,^^10^^,^^11^ Thus, NGF-responsive Trk-A-expressing sensory neurons likely contribute to THO formation. In mice, where most of this work has been done, only half of adult nociceptors express TrkA and/or TRPV1; however, in humans both receptors are expressed by all nociceptors.

Trauma-induced heterotopic ossification clinically occurs in proportion to the severity of injury. For instance total body surface area burns, sepsis, concomitant fractures/head injury/spine injuries, and presence of bacterial wound colonization confer susceptibility to THO.^12^^,^^13^ This correlation between extent of THO formation and severity of injury is thought to occur secondary to nociception and nociception-induced neural inflammation (NINI). TRPV1 is a key player in NINI, and mediates signals via the secretion of neuropeptides such as substance P (SP) and calcitonin gene-related peptide (CGRP), in response to tissue injury.^14^ SP is an undecapeptide belonging to the tachykinin family of neuropeptides, and is able to initiate expression of almost all known cytokines from macrophages and monocytes and recruits and degranulates mast cells in animal models.^6^ It is known to increase in serum collected from both FOP and THO patients.^15^ Suppression of SP with antagonist of the tachykinin receptor (NK1) suppressed THO in mice.^6^ SP is often elevated in the serum of traumatic brain injury patients and TBI-injured mice. SP can induce osteogenesis in MSCs in culture and stimulates MSCs migration in vitro,^15^ indicating that SP may be instrumental in promoting recruitment of MSCs in THO.^16^

CGRP is a 37-amino acid peptide that has 2 isoforms in the nervous system: αCGRP (produced by CALCA (an alternative splicing of the calcitonin gene—exon V) and βCGRP (produced by CALCB). They differ from each other by 1 amino acid in rodents and 3 amino acids in humans. αCGRP is the predominant form in the body, while β-CGRP is mainly produced in the enteric nervous system.^17^ For this reason our discussion in this article will mainly be focused on αCGRP, which will be referred to as CGRP for simplicity. CGRP has long been known to influence bone homeostasis and regeneration and is mainly synthesized in the dorsal root ganglia (DRG) neurons, two-thirds of which are nociceptive (high-threshold, slow conduction) Aδ fibers or C-fibers, whereas one-third is low-threshold mechanoreceptors (non-nociceptive). Synthesis of CGRP in DRG neurons is NGF-dependent, and once produced it is transported to the sensory nerve terminal where it is stored in synaptic vesicles and released in response to nociception, heat, low pH, and TRPV1 agonists such as capsaicin in animal models. CGRP causes vasodilatation, mast cell degranulation, cytokine release, neovascularization, and influences the function of both osteoblasts and osteoclasts. It also maintains homeostasis between the tissue and the sympathetic nervous system, especially as a feedback mechanism in bone for fracture pain and repair.^18^ In THO it has been directly linked to immune cell recruitment, cytokine release, and induction of osteogenic differentiation in MPCs through upregulation of cAMP levels.^19^ The CGRP receptor consists of the calcitonin receptor-like receptor (CRLR) and receptor activity modifying protein (RAMP1) in a 1:1 ratio. CRLR and RAMP1 are found on a wide variety of somatic cells including osteoprogenitor cells and osteoblasts. Once activated by binding of CGRP, RAMP1 activates multiple intracellular signaling cascades including canonical Wnt/β-catenin signaling and cAMP-cAMP-response element binding protein signaling^20^ (Figure 1).

In summary, PNS-derived SP and CGRP release into regions of injured tissue may contribute to THO formation, at least in come contexts. Both SP and CGRP are known to increase proinflammatory cytokines, recruit macrophages and mast cells, and also promote osteogenic differentiation.^21^^,^^22^ That said, these specific roles of these peptides remain to be elaborated, and they have a complex relationship in THO formation. For instance, in a murine Achilles tenotomy THO model wherein either SP, CGRP, or SP with CGRP was delivered to the tenotomy site, SP delivery alone increased THO, whereas CGRP alone had little effect. Interestingly SP treatment with CGRP mitigated the extent of THO formation, suggesting that CGRP may counteract some effects of SP in this context.^14^ In another study, genetic down-regulation of the SP receptor—Neurokinin-1 in mast cells dramatically reduced THO volume.^15^ Chemical depletion (via bisphosphonate analog—clodronate) of macrophage population; and mast cells stabilization, both independently significantly reduced THO production.^6^ Thus, nociception and NINI play an important but complex role in THO initiation.

## Role of BMPs in NINI and THO

**Figure 2:**
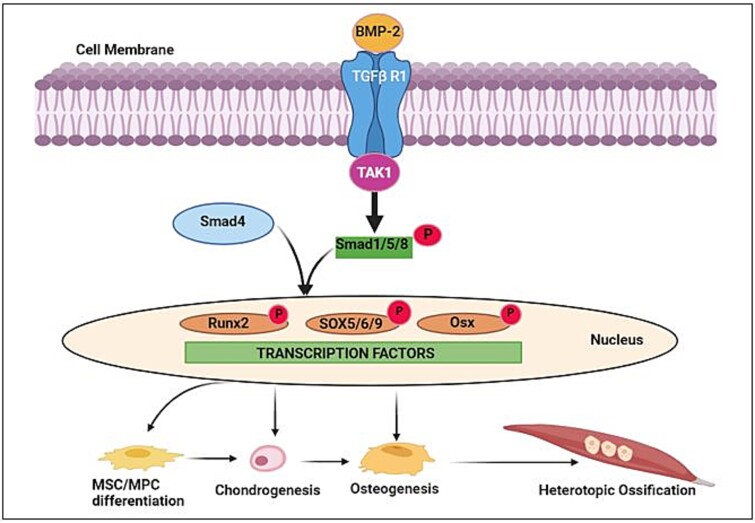
Bone morphogenic protein-2 canonical pathway in mesenchymal stem cell/mesenchymal progenitor cell (MSC/MPC) osteogenic differentiation in trauma-induced heterotopic ossification (THO). BMP-2 binds with TGFβ receptor 1 (TGFβR1) and activates TGFβ activated kinase (TAK1) leading to phosphorylation of SMAD 1/5/8. SMAD 1/5/8 complex then binds with SMAD4 and translocates to the nucleus, serving as a transcription factor for *Runx2*, *SOX5/6/9,* and *Osterix* that are critical in MSC/MPC differentiation, proliferation, chondrogenesis, and osteogenesis.

Bone morphogenic proteins were first described in 1965 and belong to the TGF-β superfamily.^23^ There are 20 identified BMPs and while their functions are diverse and still under study, they play an important role in bone and cartilage development and repair. Of these, BMP-2 is a common link found in all types of heterotopic ossification and plays an essential role in THO formation. BMP-2 is normally expressed by a variety of tissues and cells including nerves. In sensory nerves BMP-2 is produced at the nodes of Ranvier, and its expression is reduced upon nerve injury.^23^ BMP-2 upregulates both SP and CGRP in DRG in a dose-dependent manner in vivo *and* induces the release SP and CGRP by sensory nerves, which, as noted in the above section, are also released in response to noxious stimuli and by TRPV1 activation.^23^ Interestingly, TPRV1 sensory neurons have been shown to increase bone healing and TPRV1 ablation (for instance by capsaicin) delays bone healing.^24^ Tissue injury and noxious stimuli also increase levels of BMP-2 and TGFβ, both of which act via similar canonical (SMAD-dependent) and noncanonical (SMAD-independent) pathways to direct resident MSCs/MPCs down a chondrogenic/osteogenic pathway. Both SMAD-dependent and SMAD-independent pathways regulate transcription of osteogenic genes specially *Runx2* and *Osterix*. In the canonical pathway BMP-2 binds with TGFβ receptor 1 and activates TGFβ activated kinase (TAK1) leading to phosphorylation of SMAD 1/5/8. SMAD 1/5/8 complex then binds with SMAD4 and translocates to the nucleus, serving as a transcription factor for *Runx2*, *SOX5/6/9,* and *Osterix* that are critical in osteogenesis (Figure 2).^25^^,^^26^ Non-canonical BMP-2 signaling also acts via *Runx2* phosphorylation via p38 phosphorylation. Since BMPs belong to the TGFβ family, and since both BMPs and TGFβ are upregulated in sensory nerves after traumatic injury, activation of both the canonical and non-canonical pathways likely drive ectopic bone formation in tissue-resident MPCs/MSCs. Single-cell RNA-sequencing data revealed that MPCs/MSCs express high levels of TAK-1, and blocking it with a non-canonical inhibitor (NG-25) limits ectopic bone formation in a THO rat model.^11^ Previous work indicates that TAK1/TAK1 binding protein activates MKK3-P38 MAPK signaling that induces type I collagen expression by TGF-β. These pathways converge on the RUNX2 gene causing chondrogenic and osteogenic differentiation of MPCs/MSCs and appear to differentially regulate BMP2 and TGF-β function (Figure 1). Together, these studies highlight the critical role that BMP-2-signaling and downstream non-canonical activation of TAK1 play in THO initiation and progression.^27^^,^^28^

Substance P and CGRP play a synergistic role in BMP-2 induced osteogenesis.^29^ Studies on human osteoblast-like cells (MG-63) showed an increased protein expression of osteogenic markers (ALP and osteocalcin) on exposure to CGRP; however, this osteogenic differentiation was blocked if the cells were first treated with the BMP2 inhibitor Noggin before incubation with CGRP.^19^ This indicates that the BMP2 pathway is required to mediate the osteogenic differentiation by CGRP.^19^ CGRP also acts as a positive feedback mechanism in THO, being initially upregulated by BMPs and later itself upregulating BMP expression. In another study, cells that were previously virally transfected to cause overexpression of BMP-2 were injected into murine muscle. This resulted in sensory nerve activation, neuroinflammation, and release of SP and CGRP, which in turn caused mast cell recruitment to peripheral nerves.^6^ Mast cell numbers increase in the area injected with the transduced cells vs. control cells within 48 h. Degranulation of mast cells releases chymases, tryptases, and other enzymes that are essential for remodeling of the sensory nerves including sensory neurite outgrowth into the injured tissue. Blocking mast cell degranulation, on the other hand, inhibits THO formation.^6^^,^^15^^,^^30^

## Nerve-blood vessel crosstalk in THO

Nerves and blood vessels have a close relationship with each providing signals that influences the other in both bone formation (crosstalk) during embryogenesis, as well as homeostasis and fracture healing in adult bone. The mammalian skeletal system includes a dense network of nerves and blood vessels, and during embryogenesis the nerves and vessels travel into the medullary cavity to influence bone pattern formation. The intersection of neural ingrowth and angiogenesis at sites of bone formation is a common finding. TrkA inhibition led to a 30% reduction in vascularity during fracture repair and a 50% reduction of vascularity during long bones embryogenesis.^7^^,^^31^ This close nerve-vessel relationship is recapitulated in THO where sensory innervation is necessary before angiogenesis during THO formation. As mentioned above, NGF is the major neurotrophic factor that stimulates sensory neural ingrowth after severe injury and is secreted by vascular pericytes.^32^ NGF activates TrkA, facilitating sensory neural invasion and ultimately the osteogenic differentiation of MPCs/MSCs. TrkA agonism increases sensory innervation, a shift of signaling from FGF to TGF-β at the site of injury, and increased angiogenesis preceding THO formation.^10^^,^^32^ Inhibiting TrkA signaling attenuates innervation, angiogenesis, and THO formation, suggesting that innervation and subsequent angiogenesis is indispensable for THO formation and maturation.^7^^,^^10^ Furthermore, denervation leads to a loss of mesenchymal TGF-β signaling and attenuation of THO formation, implicating it as an important signaling pathway that drives THO formation. In another study, a high degree of spatial neurovascular congruency was observed in response to injury in a murine THO model.^33^ Neurectomy blunted the vascular response and attenuated key angiogenic transcriptional pathways, type H vessel formation, and endothelial proliferation. Chemical and genetic inhibition of axonal growth also led to similar deficiencies in angiogenesis and THO bone formation. Single-cell transcriptomics identified key neural-derived angiogenic factors that may mediate neurovascular signaling in THO such as FGF18, PDGF, and VEGF.^34^ However, it should be noted that no studies were performed to prove that inhibiting angiogenesis affects neural ingrowth. Additionally, each of these factors independently regulate bone formation: for instance, impaired FGF-18 signaling was shown to reduce bone formation independent of angiogenesis.^34^ VEGF is well known to regulate endochondral ossification and PDGF is the ligand for PDGFRα—a key osteogenesis regulator.^33^^,^^35^^,^^36^ Overall, neuro-vascular coupling appears to be influenced by paracrine factors such as SP, FGF, PDGF, VEGF, TGF-β, and Wingless-related integration site (Wnt).^32^ Neural VEGF is required for angiogenesis, and NGF can promote vascular growth. On the other hand, SP can regulate VEGF expression and enhance BMP-2 signaling, promoting both angiogenesis and osteogenesis.^10^^,^^32^ The principle factors that influence neural and vascular ingrowth at the site of injury, and how this in turn regulates the differentiation of MSCs/MPCs is still essentially undefined.

**Figure 3:**
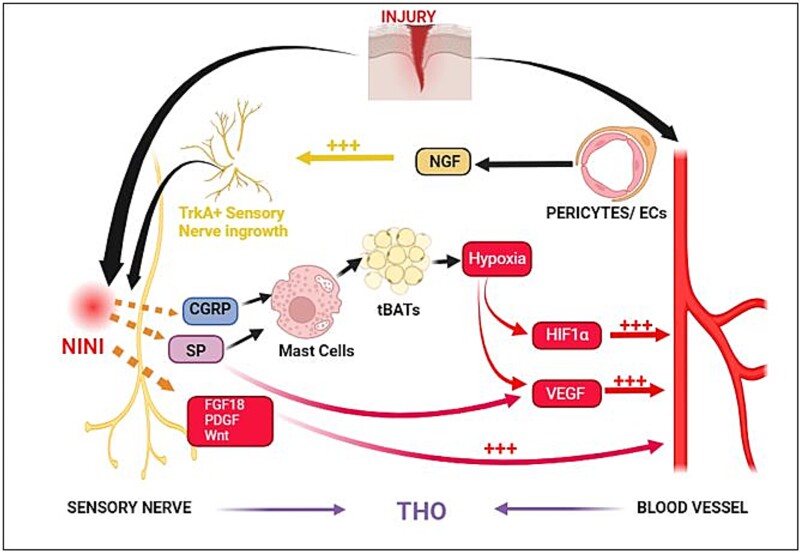
Nerve-blood vessel crosstalk in trauma-induced heterotopic ossification (THO). Injury leads to vascular pericytes and smooth muscles releasing nerve growth factor (NGF), stimulating sensory neural ingrowth and ultimately osteogenic differentiation of resident progenitor cells. Sensory nerve activation by NGF leads to nociception-induced neural inflammation (NINI) via the release of substance P (SP) and calcitonin gene-related peptide (CGRP), which cause mast cell degranulation, activation of transient brown adipose-like tissue cells (tBATs), local tissue hypoxia and ultimately VEGF and hypoxia inducible factor alpha (HIFα) expression, and angiogenesis. Activated sensory nerves also secrete FGF-18, PDGF, and wingless-related integration site (Wnt), all of which further regulate endothelial cells (ECs) to promote angiogenesis. To regulate ECs after nerve invasion to promote angiogenesis and further stimulate heterotopic ossification at the injury site.

Another interesting neuro-vascular interaction includes the transient brown adipocyte-like cells (tBATs) that have been observed in early THO formation in rodent THO-models.^37^^,^^38^ These cells express uncoupling protein 1 (UCP1) in the mitochondria and appear to be derived from nerve tissue progenitors. UCP1 uncouples ATPase from the electron transport chain causing the energy that would otherwise be utilized for ATP synthesis is released as heat. The uncoupled respiration and rapid oxygen consumption results in cellular hypoxia-like responses in the vicinity of the tBATs.^38^ There is a 70-fold increase of tBATs on day 2 after BMP-2 induction in a THO murine model dropping to baseline on day 4.^37^ Olmsted-Davis have previously shown that tBATs contribute to the reduction of oxygen tension in the microenvironment, triggering increased VEGF production and new vessel formation just prior to the cartilage formation stage of THO.^39^ Studies in mice lacking UCP1 also reduced THO formation suggesting that UCP1-associated creation of this oxygen gradient is an essential step in the early phases of THO.^9^ The hypoxia-like signals produced by tBATs could lead to the activation of hypoxia inducible factor I alpha, that in turn increases VEGF-A expression and ultimately new vessel formation within the injured tissues (Figure 3).

Salisbury et al. demonstrated in a murine model that BMP-2 delivery caused sensory nerve-induced neuroinflammation, which in turn increased mast cell degranulation that ultimately activated the sympathetic nervous system with the release of noradrenaline.^38^ Cell tracing studies showed that in response to β-adrenergic receptor activation, ADRβ3+ and UCP1+ progenitor cells within the perineurium migrated away from the nerves and into the damaged tissue and ultimately formed tBATs. Serotonin release from mast cell degranulation may aid in the production and activation of tBATs since blocking mast cell degranulation also blocked the expression of UCP1 in these progenitor cells. tBATs have also been identified in human THO tissue.^38^ These data suggest that tBATs formation is modulated by neuroinflammation, and involved with the angiogenesis that follows sensory nerve ingrowth within the THO-susceptible tissue injury site.^39^ The fate of the tBATs is still unknown, it is believed that they likely they degenerate over time, once the initial inflammation from the injury dissipates.

## Sympathetic nervous system and THO

In regular bone homeostasis, the new bone laid down by osteoblasts must replace the bone resorption by osteoclasts in order for the bone mass to be kept stable. This coupling between bone formation and resorption is driven by multiple factors including the ANS. Although the role of the ANS in bone homeostasis and fracture healing are not completely understood, the prominent presence of sympathetic nerves in areas of highest mechanical stress and metabolic turnover points to a potentially important role. The post-ganglionic neurons of the sympathetic nerves are generally noradrenergic, unmyelinated and <5 μm in diameter. In bones, they run along with the somatic sensory and motor nerve fibers as well as within the adventitia of the blood vessels supplying the bones. Bone healing animal model studies have shown that the density of sympathetic nerve in growth within the fracture site and especially in non-unions increases significantly. Limb denervation did not completely stop callus formation but did weaken the bone healing. Furthermore, pre-fracture sympathectomy using 6-hydroxydopamine in a murine model showed low callus bone volume and a mechanically weaker callus. Chemical sympathectomy with 6 hydroxy-dopamine significantly impaired the mechanical properties of uninjured and fractured bone and affected callus maturation, suggesting that sympathetic nerves play an important role in normal bone homeostasis and delay mesenchymal cell differentiation within the callus in fracture healing.^40^ Sympathetic nerves have been postulated to play a role in THO, and our group (Levi et al.) has previously noted abnormally high levels of tyrosine hydroxylase positive (TH+) nerve fiber ingrowth at the THO site.^10^ Noradrenaline released by sympathetic nerves within injured tissues stimulates β3-adrenergic receptors and increases tBATs in mice, dogs, and primates.^7^ The increased noradrenaline and adrenergic receptor expression found within the injured tissue is thought to promote sensory neurite spurting, inflammation, and neuropathic pain. The exact mechanism by which sympathetic nerves regulate THO is understudied and future studies will have to precisely define the relative importance of sensory versus sympathetic nerves.^7^ Similarly, the role of the parasympathetic system is poorly understood within the context of THO formation.

## Blood-nerve barrier and stem cells in THO

**Figure 4:**
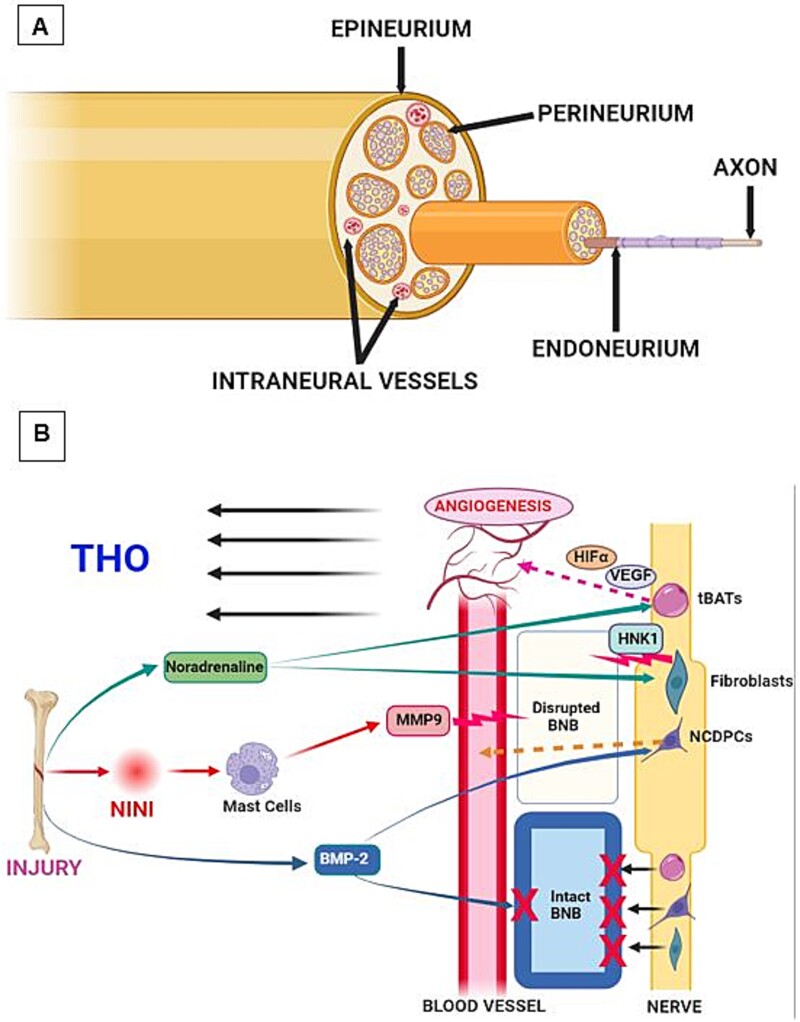
(A) Peripheral nerve anatomy and nerve-vessel crosstalk. Peripheral nerves consist of axons that are separated from the external environment by concentric layers of connective tissue. The innermost layer is the endoneurium, followed by the perineurium that binds multiple axons into fascicles, and the outermost epineurium that binds the fascicles into a nerve. Neural vessels either run on the surface within the epineurium or found within the substance of the nerve between nerve fascicles. (B) The blood-nerve barrier (BNB) consists of the endothelial cells that are bound by tight junction molecules (occluding, zona occludens, and claudin-5), which prevent large molecules such as proteins and cells to traverse into the nerve. Injury leads to pain, which leads to nociception-induced neural inflammation (NINI) and sympathetic activation by the release of noradrenaline. NINI causes mast cell degranulation and the release of matrix metalloproteinase-9 (MMP9) and histamine that drastically lowers tight junction molecules and makes the BNB leaky. This results in neural crest derived progenitor cells (NCDPCs) and transient brown adipocyte-like cells (tBATs) to leak out into the surrounding tissues. Noradrenaline activation of β-adrenergic receptors causes perineural fibroblasts to proliferate and express human natural killer-1 (HNK1) protein that further breaks down the BNB.

Peripheral nerves in vertebrates consist of axons that are separated from the external environment by concentric layers of connective tissue forming on the innermost endoneurium, surrounded by the perineurium and ultimately by the epineurium. These layers not only provide mechanical protection but also function as a barrier between the nerves, their vasculature, and the surrounding tissue. Thus, like the blood-brain barrier in the CNS, peripheral nerve axons also process a blood-nerve barrier (BNB) that separates their vasculature from the inner compartment of the nerve that contains neural crest stem cells and axons (Figure 4A). The BNB was initially described by Yosef et al. who showed that the walls of the endoneurial vessels consist of a single layer of endothelial cells that possesses tight junction molecules such as occludin, zona occludens, and especially claudin-5, that prevent large molecules such as proteins and cells to readily exit the blood vessels into the nerve, allowing for easy exchange of gas and small molecules. Neuroinflammation occurring after severe soft tissue injury drastically lowers tight junction molecules and results in opening of the BNB.^6^^,^^41^ Mast cell degranulation that occurs with neural inflammation, releases matrix metalloproteinase-9 (MMP9) that peaks within 48 h of soft tissue injury and is instrumental in the breakdown of the tight junction molecules, ultimately causing a leaky BNB.^42^ A similar increase in serum MMP9 is also noted in humans during early phases of THO.^43^ The BNB leakiness is further potentiated by the release of histamine that dramatically lowers the expression of tight junction molecules such as occludins, cadherins and claudins.^42^ Nerve-derived MPCs express claudin-5 when they cross the blood-nerve barrier into the ossification site. Finally, mast cell degranulation also activates the perineurial fibroblasts via noradrenaline activation of β-adrenergic receptors, causing these fibroblasts to proliferate. These perineural fibroblasts express the neuromigratory protein Human Natural Killer-1(HNK1) which potentially also disrupts the BNB (Figure 4B).^42^

Olmsted-Davis et al.^44^ conducted an interesting study where they injected BMP-2 producing cells into the murine quadricep muscles and noted remodeling of nerves (day 1), formation of transient brown fat (days 2-3), vessel formation (day 3), chondrogenesis (days 5-6), and immature bone formation (days 6-7). Interestingly, even though BMP-2 was injected into the muscle, the only cells that immediately responded were found within the endoneurium of nearby peripheral nerves behind the BNB. The investigators showed that within 24 h after delivery of BMP-2, neural crest derived progenitor cells (NCDPCs) within the endoneurium (ie, within the inner compartment of peripheral nerves) displayed increased markers of BMP-2/SMAD1,5,8 signaling.^44^ This signaling was BMP-2 dose-dependent and was associated with the activation of TRPV1, suggesting the involvement of nociceptor axons in the nerve. Interestingly, there were no BMP-2 effects in the soft tissues around the nerve, only within it. This lack of BMP-2 effects in the soft tissue outside the nerve was likely due to the rapid binding of BMP-2-regulating binding proteins that prevented receptor binding. Thus the BMP-2 was protected within the nerve once past the BNB, suggesting a novel mechanism by which BMP-2 selectively targets peripheral nerves to initiate THO.^44^

A significant number of cells contained within the endoneurium express osterix—an osteoblast-specific transcription factor, as well as PDGF-β—a NCDPC marker. PDGF-β is also the marker of human osteoprogenitor cells in human THO samples. Expression of osteogenic-specific transcription factors were noted in cells derived from within the endoneurium, and these transcription factors reappeared at the site of the THO formation after transiently appearing in the circulation.^45^ NCDPCs undergo an epithelial to mesenchymal transition (EMT) during embryonic osteogenesis to produce sensory nerves. Interestingly if these if these NCDPCs that are destined to form sensory nerves, are placed within the embryo, just prior to the EMT, they will form cartilage or bone.^45^ Yamanaka and Takayashi^46^ demonstrated that pluripotent cells can be created from differentiated cells through manipulation of 4 transcription factors: octamer-binding transcription factor 4 (Oct4), sex determining region Y-box 2 (Sox2), Krüppel-like family of transcription factor 4 (Klf4), and c-Myc. Sun et al. recently showed that mouse peripheral nerves contained a population of pluripotent cells that are normally quiescent, however physical (stretch, compression), chemical (blood, BMP-2), and electrical stimulation, causes a massive proliferation and egress of these cells from the nerves into surrounding tissues. These cells uniformly exhibit the 4 critical genes associated with pluripotency, ie, Oct4, Sox2, Kif4 and c-Myc. Thus, THO appears to be a recapitulation of this embryonic process of neural crest derived osteogenesis. It further appears to be associated with NCDPCs spilling out of damaged nerves through a leaky BNB and into the injured tissue to ultimately form osteoblasts and THO formation. Finally, it appears that sensory nerves relay signals to the brain regarding their environment, and it is likely THO is the result of these signals meant to replace lost and/or injured tissues have gone awry.

## Conclusion

**Figure 5:**
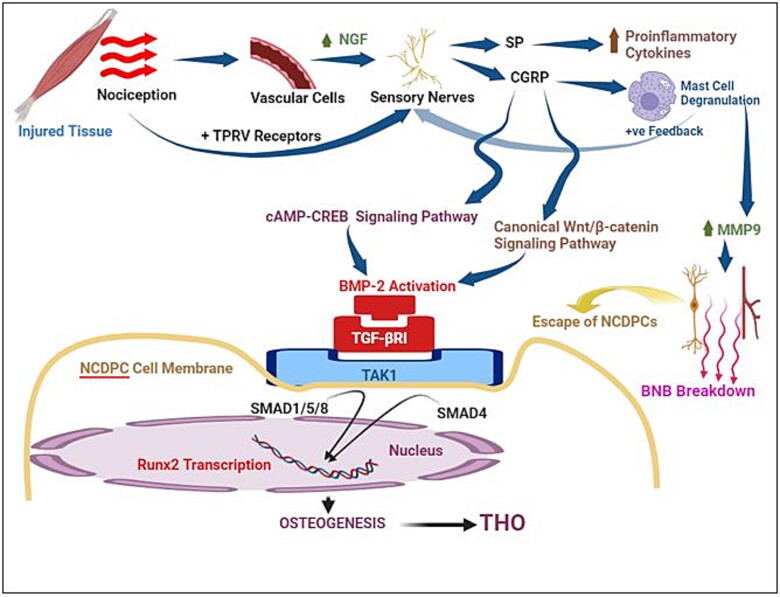
Overview of signal transduction pathways involved in nociception-induced neural inflammation (NINI) and trauma-induced heterotopic ossification (THO). NINI occurs via the release of nerve growth factor (NGF) from vascular pericytes and the ultimate release of substance P (SP) and calcitonin gene-related peptide (CGRP) from sensory nerves within the injured tissue. This leads to degranulation of mast cells, the release of matrix metalloproteinase-9 (MMP9), leading to the breakdown of the blood-nerve barrier (BNB), leading to release of neural crest derived progenitor cells (NCDPCs) into the injured tissue. Parallel to this process BMP-2 initiates the NCDPCs toward osteogenic differentiation. CGRP has direct osteogenic effects on osteoprogenitor cells/mesenchymal stem cells, by activating BMP-2 via canonical Wnt/β-catenin signaling and cAMP-cAMP-response element binding protein (CREB) signaling. BMP-2 binds to TGF-βRI and activates TGF-β-activated kinase 1 (TAK1) leading to phosphorylation of SMAD1/5/8, which binds to the co-activator SMAD4 and translocates to the nucleus to serve as transcription factor for BMP responsive genes critical in osteogenesis such as Runx2 and others.

Peripheral nerves play an integral part in THO initiation and progression as summarized below (and Figure 5):

- Tissue damage/cell death triggers nociception that increases sensory ingrowth into injured tissue.
- Injured sensor nerves release SP and CGRP resulting in neuroinflammation.
- Bone injury releases BMP-2 that selectively permeates into sensory nerves through a leaky BNB.
- NCDPCs leak through the BNB into injured tissue and are guided down the osteogenic differentiation pathway.
- Some NCDPCs differentiate into tBATs, which are responsible for the angiogenesis required for THO formation and are regulated by sympathetic nerves.

Hence, peripheral nerves provide the cell source for THO, incite NINI, and produce tBAT cells that are essential for angiogenesis and stabilization of THO, making the nerve-THO connection an important relationship that could be leveraged for THO prophylaxis and treatment. However, there are still many gaps in our knowledge of the neural involvement in THO initiation and progression. For instance, we know that sensory nerve innervation and neoangiogenesis play important roles in THO initiation and progression. Neural VEGF is required for neoangiogenesis, SP can regulate VEGF expression as well as promote BMP-2 signaling that itself can promote angiogenesis. Furthermore, NGF can also promote angiogenesis. However, the mechanism by which neuro-vascular interactions influence THO, and if they influence the differentiation pathway of MPCs/MSCs is still unclear. Whether the invading sensory neurons have a role to play in downstream signaling pathway of THO is yet to be determined. While signaling pathways commonly activated in THO such as FGF, TGF-β, PDGF, VEGF, and Wnt have long been known to play crucial roles in nerve-bone coupling during fracture healing, their link in THO is not fully elucidated. Whether or not trauma-associated sensory nerve ingrowth and neoangiogenesis are independent or interdependent processes requires studies to examine the inverse vascular-to-nerve relationship. And if they are interdependent do these interactions regulate the differentiation and proliferation of MPCs/MSCs? Finally, the role of neural inflammation in secondary THO, ie, recurrence of THO after surgical excision is also an understudied area.

Medical or surgical interventions to prevent or treat THO could in theory touch on any one of the processes outlined in this review. Reducing NINI might be one avenue to pursue to limit THO formation. Surgical denervation in rodent models of THO have previously shown to impede NINI and reduce THO formation.^24^ This is not a clinically feasible option because of the sensory loss, loss of function, and muscle atrophy in the involved limb. In amputees, surgical denervation above the zone of injury would lead to muscle atrophy and sensory loss in the residual limb and would be detrimental to prosthetic use in terms of cushioning weight bearing areas with muscle bulk. Furthermore, sensory loss limits the sensory and proprioceptive feedback required for prosthetic use.^19^^,^^47^^,^^48^ Alternatively, blocking downstream targets such as BMP and SMAD1/5/8 is an attractive approach; however these have not been successful. It is possible that that once the THO cascade is initiated, controlling the exponential progression is exceedingly difficult. Perhaps reducing upstream signaling pathways such as NINI and CGRP may be needed to thwart THO initiation. This could include clinically available inhibitors of SP such as neurokinin-1, or CGRP inhibitors (eg, Erenumab). While surgical denervation is not clinically feasible, emerging neuromodulation surgical techniques such as targeted muscle reinnervation—where amputated peripheral nerves in amputees are coapted to nearby motor branches), or regenerative peripheral nerve interface—where amputated nerves are wrapped in a muscle graft may be considered.^49^ These techniques are known to reduce pain, neural inflammation, and phantom limb pain, and may be translatable to THO. The limitation of these techniques is that they can only be used in amputees where a transected nerve is available to modulate surgically. Clinically curious phenomenon occurring with THO should also be further investigated. For instance, patients with paroxysmal sympathetic hyperactivity have frequently been noted to have concurrent THO linking it to the sympathetic system. Manipulation of the ANS (specially the sympathetic system) or upstream ANS-regulating sites within the CNS may be an interesting approach to consider.

In conclusion, the involvement of nociception and sensory nerve derived neural inflammation in triggering THO initiation is evident and the nerve-vascular crosstalk is intricately interwoven in the THO pathogenesis. However, the underlying mechanisms behind these events need to be understood before we can successfully leverage these intricate molecular events and relationships to realize their therapeutic or preventative potential. The use of neuroinflammatory markers for early diagnoses and as prognosticators should be explored, as well as therapeutics targeted toward SP, CGRP, and related pathways. The developing narrative of neural influences on THO highlights the complex interplay between sensory neurons, the sympathetic nervous system, neuroinflammatory peptides, and angiogenesis.

## Data Availability

This review article is based on published data as cited in the article and summarized in the reference section. These published articles are available in the public domain.
